# Overexpression of MicroRNA-30b Improves Adenovirus-Mediated *p53* Cancer Gene Therapy for Laryngeal Carcinoma

**DOI:** 10.3390/ijms151119729

**Published:** 2014-10-29

**Authors:** Liang Li, Binquan Wang

**Affiliations:** 1 Department of Otolaryngology, Head and Neck Surgery, the First Hospital, Shanxi Medical University, 85 South Jiefang Road, Taiyuan 030001, China; E-Mail: liliang_mu1983@163.com; 2 Shanxi Key Laboratory of Otorhinolaryngology Head and Neck Cancer, Taiyuan 030001, China; 3 Key Institute and Laboratory of Otolaryngology Affiliated with Shanxi Province, Taiyuan 030001, China; 4 Department of Otolaryngology, Head and Neck Surgery, the Second Affiliated Hospital of Nanjing Medical University, 121 Jiangjiayuan Road, Nanjing 210011, China

**Keywords:** laryngeal carcinoma, miR-30b, *p53*, field cancerization

## Abstract

MicroRNAs play important roles in laryngeal carcinoma and other cancers. However, the expression of microRNAs in paracancerous tissue has been studied less. Here, using laser capture microdissection (LCM), we detected the expression of microRNAs in paracancerous tissues. Among all down-regulated microRNAs in the center area of tumor tissues, only miR-30b expression was significantly reduced in paracancerous tissues compared to surgical margins. Therefore, to further investigate the effect of miR-30b on laryngeal carcinoma, we stably overexpressed miR-30b in laryngeal carcinoma cell line HEp-2 cells. It was found that although there was no significant difference in cell viability between miR-30b overexpressed cells and control HEp-2 cells, *p53* expression was obviously enhanced in miR-30b overexpressed cells. Whether miR-30b could improve the anti-tumor effect of adenovirus-*p53* (Ad-*p53*) in laryngeal carcinoma and other cancer cell lines was also evaluated. It was found that in miR-30b overexpressed HEp-2 cells, p53-mediated tumor cell apoptosis was obviously increased both *in vitro* and *in vivo*. MDM2-p53 interaction might be involved in miR-30b-mediated anti-tumor effect. Together, results suggested that miR-30b could modulate p53 pathway and enhance *p53* gene therapy-induced apoptosis in laryngeal carcinoma, which could provide a novel microRNA target in tumor therapy.

## 1. Introduction

Laryngeal carcinoma is one of the most aggressive cancers of the head and neck region. The survival rate of patients with laryngeal carcinoma is low due to its resistance to chemotherapy/radiotherapy and frequent local or regional recurrence after surgery excision [1,2]. Residual tumor tissue has been previously considered to be one of the main causes which induce local recurrence post-surgery [2]. However, about 3.9%–32% of patients with laryngeal carcinoma develop local recurrences although microscopically negative margins were defined [3]. A biological process called “field cancerization” has been introduced to demonstrate that a population of cells with early genetic changes (without histopathology) remains in paracancerous tissues, and was proposed to explain the locally recurrent cancer and multiple primary tumors [3,4]. Therefore, identification of the important molecular markers in these genetically transformed cells during field cancerization could be meaningful in the field of cancer therapy.

MicroRNAs (miRNAs) are small non-coding RNAs that negatively regulate the translation of messenger RNAs by binding their 30-untranslated region (30UTR) [5]. Recently, hundreds of miRNAs have been identified to possess diverse biological processes, especially in tumor researches. Some miRNAs, such as miR-125b, miR-26a and miR-24 have been found significantly down-regulated in laryngeal carcinoma and exerting anti-tumor effects [6,7,8]. However, the expression of miRNAs in paracancerous tissues and their possible relationship with field cancerization has been less investigated. In the present study, we determined the expression of some reported anti-tumor miRNAs in the paracancerous tissues of laryngeal carcinoma patients. It was found that only miR-30b was substantially decreased in paracancerous tissues compared to surgical margins among all detected miRNAs. The anti-tumor effects of miR-30b in laryngeal carcinoma and its underlying mechanisms were further studied using a HEp-2 human laryngeal carcinoma cells *in vivo* and *in vitro*.

## 2. Results and Discussion

### 2.1. MiR-30b Expression Was Down-Regulated in Paracancerous Tissue of Laryngeal Carcinoma and Overexpression of MiR-30b Improved p53 Expression via Inhibition of MDM-2

**Figure 1:**
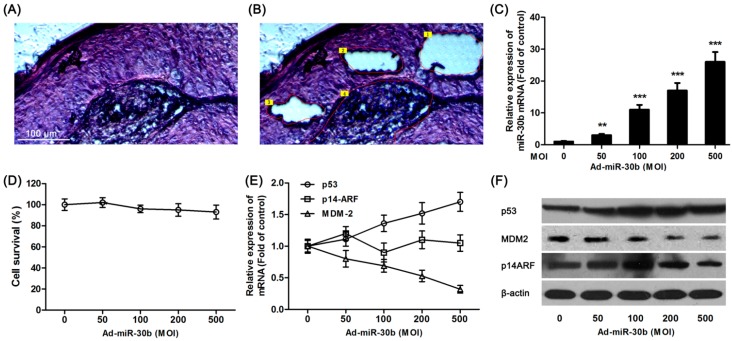
Overexpression of miR-30b regulated *p53* expression. (**A**) Hematoxylin and eosin (H&E) staining of laryngeal carcinoma sections; (**B**) Using LCM (laser capture microdissection), sections were divided into center area (Area 4) paracancerous (paraneoplastic distance <5 mm, Area 1–3) and surgical margins (paraneoplastic distance >10 mm, not shown) by LCM. Scale bar = 100 μm and referred to (**A**) and (**B**) panels; (**C**) After HEp-2 cells were incubated with miR-30-lentivirus vector for 16 h in different multiplicity of infection (MOI), the expression of miR-30b was determined using qRT-PCR. ** *p* < 0.01, *** *p* < 0.001 compared to control group, *n* = 6; (**D**) After infected by miR-30-lentivirus vector for 16 h, HEp-2 cell viability was detected by 3-(4,5-dimethyl-2-thiazolyl)-2,5-diphenyl-2-H-tetrazolium bromide (MTT) assay; (**E**) After infected, the mRNA expression level of *p53*, MDM-2 and p14-ARF were investigated using qRT-PCR; and (**F**) The expression of p53, MDM-2 and p14-ARF in protein level were detected by western blot after infected. All experiments were repeated at least three times.

Tumor tissue sections (Figure 1A) from laryngeal carcinoma patients were divided into center area (Figure 1B, Area 4), paracancerous (Figure 1B, Area 1–3) and surgical margins (not shown) by LCM. Using extracted RNA from different regions of tissue, RT-PCR results showed that in total detected microRNAs, only the expression of miR-30b was significantly down-regulated in paracancerous tissue compared with surgical margins (Table 1). In contrast, except for slightly increased expression of miR-370 and miR-16 in paracancerous tissue (not statistically significant), the expression of other microRNAs which were down-regulated in the center area of tumor changed less in paracancerous tissue (Table 1).

To further investigate the effect of miR-30b on laryngeal carcinoma, HEp-2 cells were transfected by lentiviral vector-mediated expression of miR-30b and the infection efficiency was detected by qRT-PCR (Figure 1C). As shown in Figure 1D, there was no significant effect on cell survival in miR-30b overexpressed HEp-2 cells. Meanwhile, the *p53* expression in both mRNA and protein levels was found significantly increased in infected cells (Figure 1E,F). To further investigate the expression of p14-ARF and MDM-2, which were related to the p53 pathway, western blot and RT-PCR results showed that although there was no difference in p14-ARF expression among miR-30b-overexpressed cells and the control group, MDM-2 expression was obviously down-regulated in both mRNA and protein levels (Figure 1E,F).

**Table 1:** Relative expression of microRNAs in center area and paracancerous compared with surgical margins.

MicroRNAs	Fold Change (Center Area)	Fold Change (Paracancerous)
miR-30b	0.262	0.385
miR-149	0.355	0.921
miR-26a	0.391	1.032
miR-145	0.108	0.873
miR-34a	0.514	0.932
miR-125a	0.319	0.830
miR-370	0.577	1.131
miR-203	0.110	0.779
miR-195	0.273	0.903
miR-16	0.863	1.255
miR-144	0.375	0.943

### 2.2. MiR-30b Overexpression Enhanced Ad-p53-Induced Apoptosis in Laryngeal Carcinoma and Other Tumor Cell Lines

**Figure 2:**
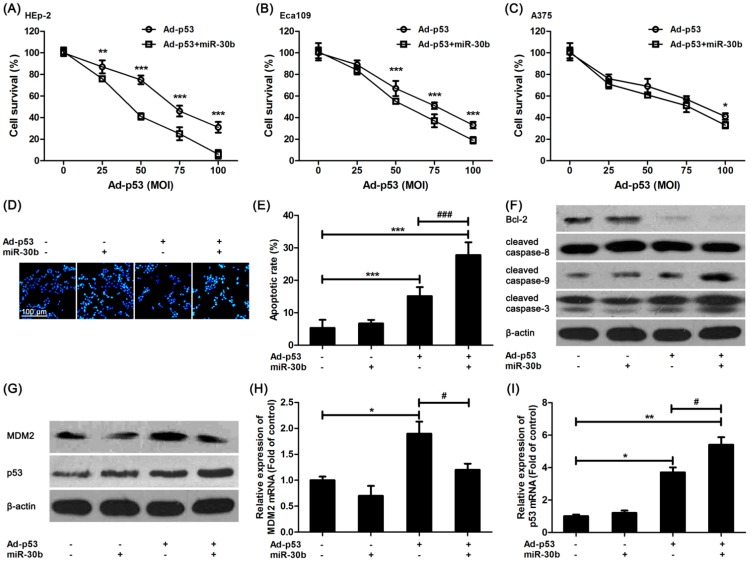
Overexpression of miR-30b improved Ad-*p53*-induced cell apoptosis via enhancing p53 pathway. (**A**) After HEp-2 cells were infected by miR-30-lentivirus vector for 16 h, Ad-*p53* vector at different MOIs was added into culture medium for another 24 h, then MTT assay was performed to detect cell viability; (**B**) Similar MTT assay was performed in Eca109 cells; (**C**) Similar MTT assay was performed in A375 cells. * *p* < 0.05, ** *p* < 0.01, *** *p* < 0.001 compared to Ad-*p53*-treated alone cells, *n* = 6; (**D**) After HEp-2 cells were infected by miR-30-lentivirus vector (200 MOI) for 16 h and stimulated with Ad-*p53* (50 MOI), the cells were stained with Hoechst33342 and bright nuclei indicated apoptotic cells. Scale bar = 100 μm and referred to all panels; (**E**) Statistical analysis of Hoechst staining, *** *p* < 0.001 compared to untreated HEp-2 cells, ^###^ *p* < 0.001 compared to Ad-*p53*-treated alone group, *n* = 4; (**F**,**G**) HEp-2 cells were treated as in Hoechst staining, western blot was performed. β-actin was used for loading control. All blots were repeated for at least three times; (**H**,**I**) HEp-2 cells were treated as in Hoechst staining, qRT-PCR was performed to detect the mRNA expression of *p53* and MDM-2. * *p* < 0.05, ** *p* < 0.01 compared to untreated HEp-2 cells, ^#^ *p* < 0.05 compared to Ad-*p53*-treated alone group, *n* = 3.

It was shown that in miR-30b overexpressed HEp-2 cells, the damage of Ad-*p53* on HEp-2 cells was increased compared to that in control HEp-2 cells (Figure 2A), and a similar result was found in another esophageal squamous cell line, Eca109 (Figure 2B). However, this intensive effect of miR-30b in A375 cells was not as efficacious as the others (Figure 2C). We further detected the Ad-*p53*-induced apoptosis in miR-30b overexpressed and normal HEp-2 cells. Figure 2D,E exhibited that the number of Ad-*p53*-induced apoptotic cells was increased obviously in miR-30b overexpressed cells in comparison with normal HEp-2 cells. Moreover, the expression of one important anti-apoptotic protein, Bcl-2, was down-regulated in Ad-*p53*-treated cells, especially in the miR-30b overexpressed group, while the caspase-3 activation level was significantly improved (Figure 2F). It was also confirmed that caspase-9 activation but not caspase-8 might be involved in this process (Figure 2F).

The expression level of MDM-2 and p53 was also detected by using western blot and RT-PCR. As shown in Figure 2G,H, MDM-2 expression was improved in Ad-*p53*-treated cells. However, the increased expression was restored in the miR-30b overexpressed group (Figure 2G,H), which was in consistence with the result in Figure 1F. As a result, there was a higher expression level of p53 in the Ad-*p53*-treated miR-30b overexpressed group (Figure 2G,I).

### 2.3. Overexpression of MiR-30b Could Improve the Sensitivity of Ad-p53 in HEp-2-Transplated Nude Mice

**Figure 3:**
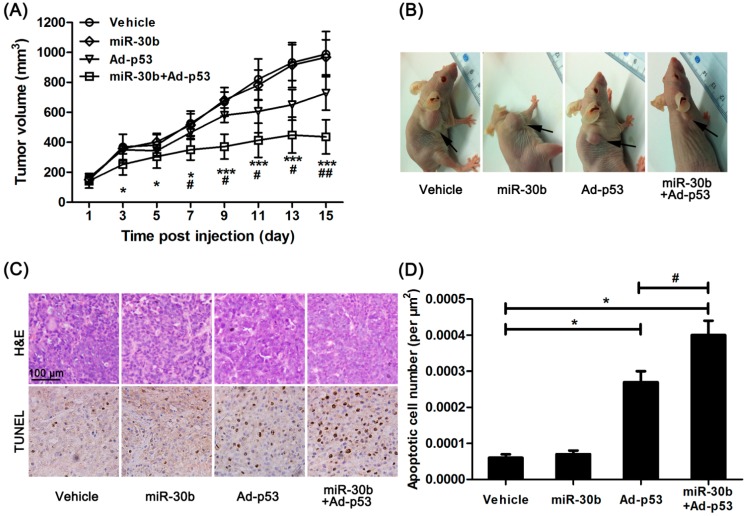
Overexpression of miR-30b improved the anti-tumor effect of Ad-*p53* *in vivo*. (**A**) After Ad-*p53* injection, tumor volume was calculated every two days, * *p* < 0.05, *** *p* < 0.005 compared to normal HEp-2 cell-implanted nude mice, ^#^ *p* < 0.05, ^##^ *p* < 0.01 compared to Ad-*p53*-treated implanted mice, *n* = 8; (**B**) After 15 days injection, the tumor in nude mice was pictured, the black arrow in the representative figure indicating tumor position; (**C**) After the experiment, the tumor tissue was removed and made into sections. H&E and TdT-mediated dUTP nick end labeling (TUNEL) staining were performed. Brown showed TUNEL-positive nuclei, and all nuclei were stained with hematoxylin. Scale bar = 100 μm and referred to all panels; and (**D**) Apoptotic cell number was calculated as TUNEL-positive number divided by total cells. * *p* < 0.05 compared to normal HEp-2 cell-implanted nude mice, ^#^ *p* < 0.05 compared to Ad-*p53*-treated implanted mice, *n* = 8.

We further investigated the effect of miR-30b on Ad-*p53*-treated HEp-2 cells *in vivo*. Normal HEp-2 and miR-30b stably infected HEp-2 cells were implanted in nude mice, and Ad-*p53* was given to mice by multiple-center intratumoral injection. After 15 days treatment, the tumor growth originating from HEp-2 cells in nude mice was inhibited by Ad-*p53* treatment, while this effect was obviously enhanced in the miR-30b overexpressed group (Figure 3A,B). Additionally, HE staining showed the presence of well-differentiated tumors with extensive necrosis in the miR-30b overexpressed group (Figure 3C). Moreover, under Ad-*p53*-treatment, the apoptotic cell number was also significantly higher in the miR-30b overexpressed group compared to normal HEp-2-implanted nude mice (Figure 3C,D).

### 2.4. Discussion

It is widely accepted that laryngeal carcinoma is a significant cause of morbidity and mortality nowadays. Because of frequently occurring local or regional recurrence and distant metastases, traditional treatments including adjuvant radiotherapy after surgery and chemotherapy could not improve survival obviously [3,9,10]. Thus, to further understand the molecular concepts of laryngeal carcinoma and search for potential therapies has been encouraging and urgent. Laryngeal carcinoma has been proven to arise from a common premalignant progenitor which has genetic alterations followed by activation of proto-oncogenes and inactivation of tumor-suppressor genes [3]. After observing abnormal epithelium in the mucosa adjacent to head and neck cell carcinoma, Slaughter *et al.* [4] had coined the term “field cancerization” since 1953. Some investigations, including microsatellite analysis and *p53* mutational, revealed the genetic alterations in the adjacent position to primary carcinoma, thus providing evidence that field cancerization could be an important factor in the recurrence of laryngeal carcinoma after therapy [11,12].

Therefore, to detect the possible genetic alterations in the cells of the surgical margins, we compared some microRNAs expression in paracancerous tissue to tumor center or normal margins of resection. Recently studies have revealed that microRNAs could be potential biomarkers in cancer research because of its unique expression profiles in tumor tissue [13]. More importantly, aberrant microRNAs expression was related to early cancerization and has been used for cancer detection and prognosis [14]. Some of them could also be possible therapeutic targets in cancers. For example, miR-24 [8], miR-34a [15] and some other microRNAs had been found significantly down-regulated in laryngeal carcinoma and could be regarded as tumor suppressors. However, the expression of microRNAs in paracancerous tissue and the relationship with field cancerization has been studied less. Using LCM technology, we isolated the paracancerous tissue, tumor center and surgical margins and detected the expression of some reported tumor suppressor microRNAs. We found that only miR-30b was still significantly down-regulated not only in tumor tissue but also in paracancerous tissue among all detected microRNAs. 

Thus, further studies were performed to investigate the possible effect of miR-30b in laryngeal carcinoma HEp-2 cell line. miR-30b has been reported to affect lactation and involution in the developing mouse mammary gland [16]. Zhu *et al.* [17] has also reported that miR-30b could increase apoptosis in gastric cancer cells. How miR-30b is integrated into tumor cell apoptosis remains unknown. In this study, we found overexpression of miR-30b in HEp-2 cells could increase the expression of *p53*, one important tumor suppressor gene [18]. In this process, MDM2, a p53-specific E3 ubiquitin ligase which could promote p53 degradation [19], was down-regulated in miR-30b overexpressed HEp-2 cells. However, using an online microRNA target prediction tool (Target Scan Human 6.2), it was found that there were fewer matched nucleotides between miR-30b and MDM2. The luciferase reporter assay of 3'-UTR was also constructed to verify the direct binding of miR-30b followed these comments. Disappointingly, the 3'-UTR of MDM2 does not respond to miR-30b (Data was not shown). Meanwhile, there was no different expression of a MDM2 inhibitor p14-ARF between each group. We speculated that miR-30b could affect MDM2-p53 interaction to indirectly regulate MDM2 and *p53* expression [19], or another binding site of miR-30b might exist in the open reading frame of MDM2 [20]. Together, results inferred that miR-30b might affect MDM2-p53 interaction, thereby participating in apoptotic process.

However, overexpression of miR-30b has less effect on control HEp-2 cells. Insufficient *p53* expression might explain this appearance. As miR-30b could inhibit MDM2 expression, therefore, we investigated whether overexpression of miR-30b could enhance the effect of adenoviral *p53* gene therapy, which has been used in clinical cancer therapy [21]. As expected, the pro-apoptotic effect of Ad-*p53* was obviously enhanced in miR-30b-overexpression laryngeal carcinoma cell line HEp-2 and another esophageal cancer cell, Eca109. However, the intensive pro-apoptotic effect in A375 human amelanotic melanoma cells was not as efficient as in HEp-2 and Eca109 cells. The different p53 pathway might contribute to this result [22]. To further investigate the Ad-*p53*-induced cell apoptosis process, mitochondrial apoptosis-associated proteins were detected in this study. Several evidences suggested that p53 signals leads to regulation of Bcl-2 expression and caspase-9-associated mitochondrial apoptosis [23,24]. We found that overexpression of miR-30b obviously enhanced Ad-*p53*-induced apoptosis via caspase-9 activation but not caspase-8. The expression of anti-apoptotic protein Bcl-2 was also inhibited in Ad-*p53*-treated miR-30b overexpression cells. To further investigate the possible effect of miR-30b on anti-tumor therapy *in vivo*, we compared the effect of Ad-*p53* on normal HEp-2 cells and miR-30b stably overexpressed cells implanted in nude mice. It was also proven that overexpression of miR-30b could significantly enhance the anti-tumor and pro-apoptotic effect of Ad-*p53* in HEp-2 implanted nude mice.

## 3. Experimental Section

### 3.1. Tissue Preparation for LCM, RNA Isolation and Quantitative Real-Time PCR

Patients with oral and oropharyngeal carcinoma were selected and divided into different groups according to Tumor Node Metastasis (TNM) staging from the 2012–2013 files of the department of otolaryngology in the First Hospital of Shanxi Medical University, all of whom gave their written informed consent to the study. The patients without lymph node metastasis were selected in this study (T3N0M0, seven patients, five male and two female). After surgical tissue specimens were collected and frozen in liquid nitrogen immediately, the 10 μm serial frozen sections were made and then hematoxylin-eosin (HE) staining was performed. Isolation of laryngeal carcinoma, paracancerous tissue and normal cells in surgical margins was performed by LCM, as described previously. [25]. At least 5000 cells in each captured tissues were used to isolate total RNAs using RNA Isolation Kit (Stratagene, La Jolla, CA, USA) following its protocol. To obtain sufficient RNA for quantitative real-time PCR (qRT-PCR), RNA amplification was performed using the MessageAmp Kit (Ambion, Foster City, CA, USA). A human universal reference RNA was also amplified in the same way.

The amplified RNA was reversely transcribed into cDNA, and qRT-PCR was performed to detect gene expression on ABI 7900 (ABI, New York, NY, USA) using SYBR Green Real-Time PCR Kit (Takara, Otsu, Japan) as described previously [26]. The primers used were: *p53*, F: TCAACAAGATGTTTTHNSCCCAACTG, R: ATGTHNSCCTGTGACTHNSCCTTGTAGATG; p14-ARF, F: GTTTTCGTGGTTCACATCCC, R: ACCAGCGTGTCCAGGAAG; MDM-2, F: TGTGTGAGCTGAGGGAGATG, R: CACTTACGCCATCGTCAAGA; β-actin, F: AATGTCHNSCCGGAGGACTTTGAT, R: AGGATGHNSCCAAGGGACTTCCTG; U6, F: GCTTCGGCAGCACATATACTAAAAT, R: CGCTTCACGAATTTGCGTGTCAT, respectively. Other primers used for microRNAs were listed in Table 1, microRNAs were normalized with U6 and other genes were normalized with GAPDH.

### 3.2. Cell Culture and Stably Overexpression of MiR-30b

Human laryngeal carcinoma Hep-2 cells and A375 human amelanotic melanoma cells were obtained from the American Type Culture Collection (ATCC, Manassas, VA, USA). Human esophageal squamous Eca109 cells were from Chinese Academy of Science (Shanghai, China). Cells were maintained in DMEM supplemented with 10% fetal bovine serum at 37 °C with 5% CO_2_. Lentiviral cloning with miR-30b purchased from System Biosciences (Mountain View, CA, USA) were used to create stable cell lines overexpression miR-30b following its approach. Different multiplicity of infection (MOI, 0, 50, 100, 200, 500) of lentivirus was added to HEp-2 cells. After 16 h incubation, MTT assay was used to detect cell survival of HEp-2, western blot and qRT-PCR were performed to detect the expression of *p53*, MDM-2 and p14-ARF in the protein and mRNA level.

### 3.3. MTT ASSAY, Hoechst Staining, Western Blot and qRT-PCR Assay

For 3-(4,5-dimethyl-2-thiazolyl)-2,5-diphenyl-2-H-tetrazolium bromide (MTT) assay, cells were seeded in 96-well plates (10,000 cells per well) and treated with/without 200 MOI of lentivirus for 16 h, followed by different MOI of Ad-*p53* (SiBiono GeneTech, Shenzhen, China, control group received Ad-*LacZ*) for 24 h. Then the cell viability was determined by MTT reagent as described previously [27].

For Hoechst staining, HEp-2 cells were cultured on cover glasses in 12-well plates and incubated with 200 MOI of lentivirus followed by Ad-*p53* (50 MOI, control group received Ad-*LacZ*) treatment for 24 h. The apoptotic cells were detected by Hoechst33342 staining as described [27]. The pictures were taken by DP70 fluorescent microscope (Olympus, Tokyo, Japan). The apoptotic ratio was calculated as apoptotic cells divided by total cell number.

After treatment as Hoechst staining, HEp-2 cells were lysed by protein extraction kit (Beyotime, Shanghai, China) according to the protocol. Western blot was performed as previously described [27]. All primary antibodies were purchased from Cell Signaling (Danvers, MA, USA), the dilutions were: *p53* (1:500), MDM-2 (1:1000), p14-ARF (1:1000), cleaved caspase-3 (1:1000), Bcl-2 (1:500), cleaved caspase-8 (1:1000), cleaved caspase-9 (1:1000), β-actin (1:5000). All blots were performed at least three times; the optical density was analyzed with Quantity One software. Cells were lysed by Trizol (Takara) for mRNA isolation, qRT-PCR was also performed to detect *p53* and MDM-2 expression in mRNA level as described above.

### 3.4. Animal Models

Six-week-old female nude BALB/c mice (20~22 g) were used in this experiment. A cell suspension of normal HEp-2 cells or miR-30b stably overexpressed HEp-2 cells was prepared (1 × 10^8^ cells/mL). A total 1 mL suspension was subcutaneous injected into the right upper flank of mice. When tumor mass reached 50 mm in diameter, implanted mice received multiple intratumoral injection of Ad-*p53* or Ad-*LacZ* (1 × 10^10^ pfu/100 μL) twice weekly. Animals were observed daily and tumor volume was recorded every two days. The tumor volume was calculated as (short diameters^2^ × long diameters)/2. Fifteen days after administration, all mice were sacrificed and tumor tissues were removed for continuous experiments. All studies were permitted by the committee for animal experiments of the first hospital of Shanxi medical university.

### 3.5. Immunohistochemistry

Hematoxylin and eosin (H&E) and TUNEL staining were performed as previously described [28]. Briefly, tumor tissues were frozen in opti-mum cutting temperature compound (OCT, Beyotime, Shanghai, China) and made to 10 μm frozen sections. H&E stain were performed to observe the morphology of tissues. Apoptotic cells were determined by TUNEL kit (Roche, Indianapolis, IN, USA) followed its protocol in tumor tissues. The apoptotic rate was calculated as TUNEL-positive cells divided by total cells. All figures were captured by microscopy (DP70, Olympus, Tokyo, Japan).

### 3.6. Statistical Analysis

All data were record as mean ± SE. Difference between groups was analyzed using two-sided *t* test, *p* < 0.05 was considered significant.

## 4. Conclusions

Collectively, this study showed that miR-30b was significantly down-regulated in paracancerous tissue of laryngeal carcinoma, which may be helpful in identifying the risk of local recurrences after surgical resection. Furthermore, forced expression of miR-30b substantially improved tumor suppressor gene *p53* expression via inhibition of MDM2, which also significantly enhanced the effect of Ad-*p53* gene therapy, suggesting that miR-30b might be a novel therapeutic strategy for the treatment of laryngeal carcinoma.
